# Cell wall deficiency as an escape mechanism from phage infection

**DOI:** 10.1098/rsob.210199

**Published:** 2021-09-01

**Authors:** Véronique Ongenae, Ariane Briegel, Dennis Claessen

**Affiliations:** ^1^ Molecular Biotechnology, Institute of Biology, Leiden University, PO Box 9505, 2300 RA Leiden, The Netherlands; ^2^ Centre for Microbial Cell Biology, Leiden University, Leiden, The Netherlands

**Keywords:** bacteriophages, resistance, cell wall, l-forms, cell wall deficiency

## Abstract

The cell wall plays a central role in protecting bacteria from some environmental stresses, but not against all. In fact, in some cases, an elaborate cell envelope may even render the cell more vulnerable. For example, it contains molecules or complexes that bacteriophages recognize as the first step of host invasion, such as proteins and sugars, or cell appendages such as pili or flagella. In order to counteract phages, bacteria have evolved multiple escape mechanisms, such as restriction-modification, abortive infection, CRISPR/Cas systems or phage inhibitors. In this perspective review, we present the hypothesis that bacteria may have additional means to escape phage attack. Some bacteria are known to be able to shed their cell wall in response to environmental stresses, yielding cells that transiently lack a cell wall. In this wall-less state, the bacteria may be temporarily protected against phages, since they lack the essential entities that are necessary for phage binding and infection. Given that cell wall deficiency can be triggered by clinically administered antibiotics, phage escape could be an unwanted consequence that limits the use of phage therapy for treating stubborn infections.

## Graphical abstract

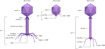

## 1. Introduction

Bacteriophages, or (in short) phages, are viruses that infect bacteria. It has been estimated that they outnumber bacteria in the biosphere by a factor of 10 and are present in almost all natural environments [1,2]. As phages are non-motile microorganisms, it is presumed that the initial contact between a phage and a suitable host occurs via random collisions as a result of free diffusion [3]. Phages recognize their host species by interacting with specific receptors, especially sugars and proteins, exposed on the bacterial cell surface. Here, we propose that the ability of bacteria to shed their wall may be an underappreciated mechanism to evade phage infection, as phages may no longer be able to recognize their host.

In this perspective review, we will start with discussing the detailed structure of the bacterial cell envelope and its specific components that enable phage attachment to their hosts. Currently, all known mechanisms for this first step of host infection involve bacterial surface-associated macromolecules. We then discuss the ability of several bacteria to shed their cell wall under influence of stressors and the consequences of a cell wall-deficient (CWD) lifestyle. Finally, we will discuss how phage infection may be evaded by shedding of the cell wall. We hypothesize that phages will no longer be able to recognize their bacterial host and render such wall-less cells immune to phage infection. Since cell wall-less states have been reported to occur in pathogenic species [4,5], this evasion mechanism may be highly relevant for the development of phage therapy treatments.

## 2. Phage–host attachment

**Figure 1.**
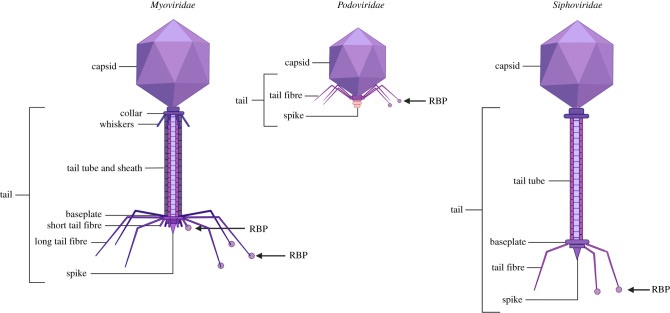
Tailed bacteriophages. The *Caudovirales* order consist of three families: (*a*) *Myoviridae*, with a contractile tail, (*b*) *Podoviridae*, which have no baseplate and are short-tailed and (*c*) *Siphoviridae* with a long non-contractile tail. RBPs can be found on long- or short-tail fibres and sometimes even on the spike. Created with BioRender.com.

Bacteriophages used to be classified according to morphological types, while more recently, sequence similarity and phylogenetic relationships have become the primary method to distinguish taxa [6,7]. The majority of phages contain double-stranded DNA in their capsid heads, although single-stranded DNA, single-stranded RNA and even double-stranded RNA are also common in nature [8–10]. Approximately 96% of all known phages are tailed phages (figure 1) belonging to the order *Caudovirales*, which can vary tremendously in size, structure and DNA content. This order can be sub-divided into three families, based on the contractility of the tail, whereby phages with non-contractile tails can be divided into those with short and long tails [7,11]. The best-studied organism in this order is the bacteriophage T4. This *Myoviridae* is well studied by cryo-EM, and detailed information on the structure and infection process of its host *Escherichia coli* are available [12–14].

**Figure 2.**
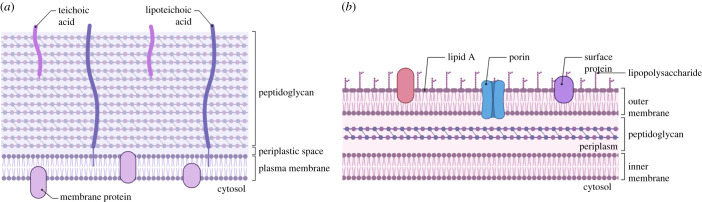
Bacterial cell wall. (*a*) The cell wall of monoderm bacteria consists of a thick peptidoglycan layer intertwined with teichoic acid, which can both be recognized by RBPs of phages. (*b*) Diderm bacteria have a thinner peptidoglycan layer and an additional outer membrane with several components that phages can recognize for attachment. Created with BioRender.com.

Although phages come in various shapes and sizes, one common feature is the need to identify and attach to a suitable host. The receptor-binding proteins (RBPs), often located at the tip of the phage's tail, recognize surface-associated molecules on a host bacterium. The nature of the bacterial molecules recognized by phages differs between various taxonomic groups and is commonly highly specific for the host cell wall or cell envelope composition. A major component of the bacterial cell wall is peptidoglycan, which is often associated with phage adsorption in monoderms (formerly called Gram-positive bacteria) [15]. Peptidoglycan strands are composed of chains of monomers consisting of N-acetylglucosamine and N-acetylmuramic acid, which are covalently cross-linked via peptide stems to create a gigantic molecule, called the sacculus [16]. Besides peptidoglycan, other macromolecules can be found in the cell wall of monoderm bacteria (figure 2*a*), such as teichoic acids, which are also known to be involved in phage adsorption [17]. In fact, over 96% of the molecules identified on monoderm bacteria so far are associated with either residues or structures of teichoic acid and peptidoglycan [18]. Diderm (Gram-negative) bacteria on the other hand display a large variety of molecules, as seen in figure 2*b* [12]. Molecules that can be recognized by phages are for instance, enzymes, transporter proteins, substrate receptors and many more structures located on the outer membrane [19]. Multiple studies have used the model organism *E. coli* and more recently also *Salmonella* to identify new molecules that are necessary for phage infection in diderm bacteria, such as the important proteins OmpA, OmpC, OmpF and LamB, but also many lipopolysaccharides [20–24].

In addition to receptors molecules, proteins and lipopolysaccharides, some phages can also attach to structures that are not located directly on the cell wall or outer membrane of bacteria, such as flagella, pili and capsules. The adhesion of phages to a flagellum often starts as a process that initially is reversible. However, the rotating movement of the flagellum guides the phages towards the cell body, where it then tightly attaches to surface molecules located near the base of the flagellum [18,25]. In addition, some phages can ‘hitchhike’ on non-host bacterial flagella to increase their chances of meeting a host species. This has been shown for the bacteriophage PHH01 that can attach to the flagella of the carrier bacteria *Bacillus cereus* to help encounter its host *E. coli* [26]. Another recent example is the transport of *E. coli* lambda phage by *Capnocytophaga gingivalis* swarm [27]. Phages can also attach to extracellular structures such as pili or fimbriae, which are proteins that help bacteria to attach to each other or surfaces. For phages ϕCb13 and ϕCbK, it has been shown that the initial attachment to pili is necessary for successful attachment to *Caulobacter crescentus*, as mutant bacteria without pili could not be infected [25]. These studies demonstrate that loosing structures like pili or flagella is an effective defence mechanism from bacteria to evade phage attachment. It further suggests that the loss of the entire cell wall may be an additional effective mechanism for phage evasion.

## 3. Bacterial defence mechanisms against phages

To prevent phage infection, bacteria have developed multiple and impressive anti-phage strategies. Restriction-modification systems act as the prokaryotic innate immune system, consisting of a restriction nuclease and methyltransferase. Bacteria prevent self-cleavage by methylation of their own DNA, while incoming phage DNA is generally not methylated [28]. A more costly defence system is called the abortive infection process, where the infected cell initiates programmed cell death before the phage can replicate, thereby protecting other cells in the bacterial colony [29]. The toxin-antitoxin system can be seen as one of the components of abortive infection and consists of a toxin that inhibits bacterial cell growth upon phage infection and an antitoxin, which protects the cell during normal conditions [30]. The phage growth limitation (Pgl) system is often described as an inverted restriction-modification system or abortive infection-like mechanism, where the primary infected bacteria do not survive the infection, but mark the progeny phage DNA by methylation [31,32]. If such modified phages subsequently infect neighbouring Pgl+ bacteria, the phage is restricted. Although Pgl systems are only found in *Actinomycetes*, a comparable mechanism, called the BacteRiophage EXclusion (BREX) system was discovered in *Bacillus cereus* [33]. This system possesses two genes that are homologous to the Pgl system. However, unlike Pgl, the BREX system prevents initial phage replication by methylation [32]. In addition to protein-mediated anti-phage defences, some streptomyces species can use a chemical defence mechanism. Here, small molecules are produced that serve as phage inhibitors [34]. The only known adaptive immune system in prokaryotes is the CRISPR/Cas defence mechanism, which protects against phages and other foreign genetic elements, like plasmids and transposons [35]. Recently, a new counter-defence mechanism against CRISPR/Cas has been uncovered, where phage T4 restores the broken genomic material, thereby making progeny phages resistant against future CRISPR attacks [36]. For those interested in more detailed information on anti-phage systems and the phage-bacteria arms race, we refer to some excellent recent reviews [31,35,37,38].

Bearing anti-phage systems is associated with a trade-off between fitness costs and the benefit of resisting phages [39]. As bacteria typically only carry a small subset of the available defence mechanisms, the ‘pan-immune system’ was recently proposed [37]. Here, the authors suggest that although a single bacterial strain cannot carry all existing defence mechanisms, it can use horizontal gene transfer to access defence mechanisms encoded by closely related species.

However, the bacterial cell wall remains the first barrier a phage needs to overcome during host infection. The prime defence of bacteria is therefore directed at the initial prevention of phage attachment to the bacterial surface-associated macromolecules. When bacteria are living in a biofilm, this mode of growth already acts as the first layer of protection. Amyloid fibre networks inhibit phage transport into the biofilm and also coat the surface of individual cells to prevent phage attachment [40]. Another defence strategy used by the diderm bacterium *Vibrio cholerae* is the production of outer membrane vesicles with an almost identical cell surface as the bacteria itself, to serve as natural decoys against phages [41].

Other common mechanisms to prevent phage attachment are shielding surface-associated molecules by glycosylation, masking them with polysaccharide capsules or mutating the molecules on the bacterial cell surface [42–44]. If phages cannot recognize and attach to the molecules on the cell surface of bacteria, the host will remain uninfected. However, the production of a capsule or adaptation of receptor molecules can be metabolically expensive [45]. Therefore, a possible energetically more favourable way to escape phage binding would be by shedding the cell wall altogether. In this case, not only the molecules located on the bacterial cell surface will vanish, but also structures like the capsule, flagella and pili are no longer present.

## 4. Cell wall deficiency as a possible mechanism to escape phage attack

Some filamentous actinobacteria have the ability to shed their cell wall under influences of hyperosmotic stress or limited oxygen availability, which may be common in soil ecosystems [46]. Under these conditions, CWD cells are extruded from the vegetative mycelia. These cells are unable to proliferate without their wall and will ultimately revert to the mycelial mode of growth [47]. However, if these CWD cells acquire mutations due to prolonged exposure to hyperosmotic stress conditions, L-forms are formed that can proliferate without a cell wall [48,49]. To start a CWD lifestyle, cells first have to escape the sacculus, followed by coping with the increased oxidative stresses in the environment [47]. Thereafter, an upregulation of membrane synthesis is required, resulting in an enlarged surface area to volume ratio [50]. Not only filamentous actinobacteria, but many other bacteria, including both monoderm and diderm species, are able to switch to a CWD state. CWD cells of different species have been found in the urine of patients suffering from recurrent urinary tract infection [51]. Additionally, such CWD cells have been detected in the biofilms of individuals with chronic and aggressive periodontitis [5]. These studies support the assumption that CWD bacteria could hide inside their host for an extended amount of time, as they are insensitive to wall-targeting antibiotics and can possibly also evade elements of the host's immune system. In fact, exposure to cell wall-targeting antibiotics could even promote the formation of CWD cells. This response is best studied for β-lactam antibiotics like penicillin, which targets the catalytic enzymes involved in cell wall synthesis, resulting in lysis of the bacterial cell wall of for instance *E. coli* [52]. Prolonged exposure to these antibiotics can be used to induce cell wall deficiency in an osmotically stable environment [52]. While cells without their wall are inherently fragile, wall deficiency probably provides bacteria with some crucial advantages over walled bacteria. In their natural environment, bacteria are challenged by various stresses, such as changing environmental conditions, competing organisms and the possibility of phage attack. The formation of CWD cells has been observed as a response to escape various environmental stresses [47]. For example in *Mycobacterium bovis*, where L-forms were isolated after nutrient starvation and cryogenic stress treatments [53] or in *E. coli,* which could survive lethal treatments like autoclaving and boiling by shedding their cell wall [54]. This indicates that cell wall deficiency could be a temporary coping strategy to protect the bacteria during unfavourable environmental conditions, like the possibility of phage infection.

**Figure 3.**
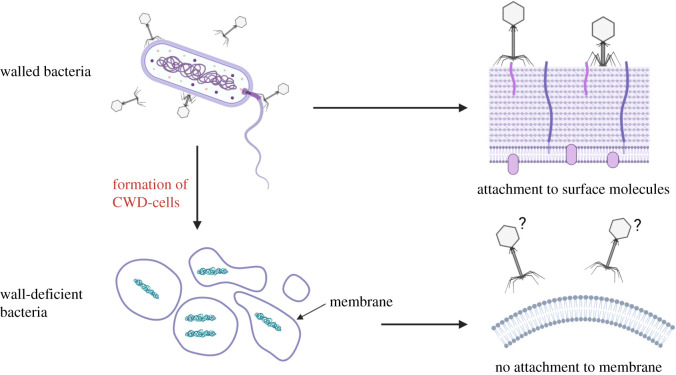
Cell wall deficiency as an escape mechanism for phage infection. Walled bacteria are susceptible to phage infection, since phages can recognize molecules located on the cell surface or bind to structures such as pili and flagella. Several bacteria can shed their wall and form wall-deficient cells. We hypothesize that phages can no longer recognize its host in this wall-deficient state, which therefore stays uninfected. Created with BioRender.com.

As the vast majority of molecules phages can attach to, are located in or associated with the cell wall, transient loss of the wall could render bacteria immune for phage binding and infection (figure 3). In monoderm CWD cells, the shedding of the wall would result in the loss of almost all known molecules a phage can recognize, as these are almost invariably associated with peptidoglycan and teichoic acids in the cell wall. The formation of diderm CWD cells is less well understood. For example, it remains debatable whether diderm bacteria that can form CWD cells retain their outer membrane, which contains the majority of the known molecules that phages recognize. Literature of CWD diderms with and without their outer membrane have been reported, and even thin-section electron microscopy cannot clearly distinguish between a single membrane and two membranes if the periplasm is pressed closely together [55,56]. On the other hand, more recent papers have shown that a rigid outer membrane is essential for the formation and survival of wall-deficient cells in *E. coli* [57,58]. Here, the outer membrane proteins Lpp, OmpA and Pal are necessary for this rigidity. These and many more outer membrane proteins could also serve as molecules for phage attachment and, perhaps in this manner, serve as a sink for phages. In this way, such CWD cells could help to protect the other cells in the colony similar to the previously described decoy outer membrane vesicles of *V. cholera* [41]. Additionally, when bacteria shift to a CWD lifestyle, small fragments of the original cell wall probably remain in close proximity of the newly formed CWD cells for some time. It may be that these small cell wall particles can also serve as a decoy for phages, just like outer membrane vesicles [41]. Building upon the theory that cell wall deficiency could be an escape mechanism for phage attack, the holin-endolysin system might also play an important role. Lytic phages use endolysins to degrade the peptidoglycan layer from inside a bacterium, which have a similar outcome as penicillin treatment. We know that penicillin is often used to induce cell wall deficiency, so possibly the holin-endolysin enzymes could be a cue for other nearby bacteria to shed their cell wall and therefore be protected from the released phages.

Wall-deficient cells of actinomycetes that revert back to filamentous growth often show a wide range of phenotypically different colonies, which can be explained by gross genetic changes [49,59]. Such mutations can sometimes lead to the evolution of beneficial traits, such as phage-resistance [60]. Many phages can infect members of the bacterial Actinobacteria phylum and for most bacteria, even multiple phages are known [61]. There is rich literature on phages that can infect Actinobacteria but, to our knowledge, no phage is currently known to infect *Kitasatospora viridifaciens*, which easily makes CWD cells. In fact, one of the features by which *Kitasatospora* species are classified in their unique genus is their resistance to the most common *Streptomyces* phages [62,63]. Perhaps *K. viridifaciens* has accumulated a specific set of mutations during many switches of CWD cells to the mycelial mode of growth in nature, resulting in broad resistance against most common *Streptomyces* phages. Nevertheless, this resistance to phages can also be explained by our hypothesis that the formation of wall-deficient cells is an escape mechanism by itself, although both possibilities are not mutually exclusive. On the other hand, switching to a wall-less state may not only be beneficial for the bacteria, but also for the bacteriophage. A recent study demonstrated that T4-like phages could be adsorbed by *E. coli* L-forms, which however did not result in lysis of these CWD bacteria [64]. These results imply that cell wall deficiency could not only be an escape mechanism for the bacterial host, but the phage may also profit from this situation, as they could switch to a pseudolysogenic lifestyle within CWD cells. Taken together, these examples demonstrate the advantages of switching to a CWD lifestyle during the possibility of phage attack.

## 5. Concluding remarks and outlook

Our knowledge about the biology of bacteriophages and their bacterial host have increased significantly over the last few decades. However, the understanding of CWD cells and their ecological role in association with phages is largely unexplored territory. The hypothesis that cell wall deficiency could be a new and underexplored mechanism for phage evasion raises several interesting research options. The most interesting one would be to investigate if phages indeed cannot recognize their usual host and whether this is due to the absence of molecules in the cell wall. On the other hand, if phages are still able to recognize their host without a cell wall, to which molecule(s) do they attach and can they eject their genome?

Perhaps CWD cells are currently one step ahead in the ongoing evolutionary arms race between phages and their host. If we assume this to be true, would there already be a sign of coevolution between the phage's RPBs and the CWD surface-associated molecules? Most research about CWD bacteria, such as mycoplasma, protoplasts and L-forms, and their interaction with phages, date from the pre-molecular era [65–67]. It would be interesting to explore their interaction with more modern technologies, such as cryo-EM, next-generation sequencing or genome editing.

Apart from exploring possible phage–host interactions and defence mechanisms in CWD bacteria, improving our understanding on bacterial and phage biology may provide biotechnological advances in, for example, phage therapy. In this case, specific phages are used as an alternative for antibiotics to treat bacterial infections [68]. Phage therapy therefore seems like a suitable strategy to treat recurrent infections and chronic diseases caused by CWD bacteria, as they are insensitive to wall-targeting antibiotics and phages can selectively kill pathogenic bacteria without harming the host or its microbiome. However, the use of bacteriophages for treating stubborn infection might be restricted if phages do not recognize wall-deficient pathogenic bacteria. In addition, clinically administered antibiotics can trigger the formation of CWD cells, which could be an unwanted consequence that limits the use of bacteriophage treatment afterwards. If phages are unable to recognize the CWD bacteria, a mixture of different phages should be considered for phage therapy or the genomes of bacteriophages could be genetically modified [69,70]. Further studies should continue to contribute valuable insights on phage infections of CWD cells and phage therapy.

## Data accessibility

This article has no additional data.
