# Correction to: ‘Cell wall deficiency as an escape mechanism from phage infection’ (2022) by Ongenae *et al.*

**DOI:** 10.1098/rsob.220199

**Published:** 2022-07-27

**Authors:** Véronique Ongenae, Ariane Briegel, Dennis Claessen

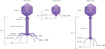

*Open. Biol.* **11**, 210199. (Published online 1 September 2021). (https://doi.org/10.1098/rsob.210199)

**Figure 1.**
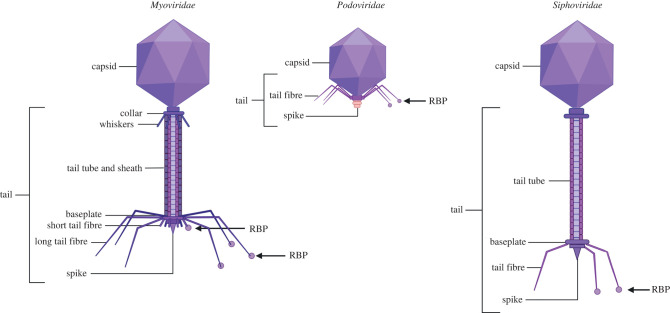
Tailed bacteriophages. The *Caudovirales* order consist of three families: (*a*) *Myoviridae*, with a contractile tail, (*b*) *Podoviridae*, which have no baseplate and are short-tailed and (*c*) *Siphoviridae* with a long non-contractile tail. RBPs can be found on long- or short-tail fibres and sometimes even on the spike. Created with BioRender.com.

The *Myoviridae* in figure 1 in our original article was missing a sheath. This has been corrected on the publisher's website.

